# *Bartonella* spp. Infection Rate and *B. grahamii* in Ticks

**DOI:** 10.3201/eid1810.120390

**Published:** 2012-10

**Authors:** Elisabeth Janecek, Andreas Mietze, Ralph Goethe, Thomas Schnieder, Christina Strube

**Affiliations:** University of Veterinary Medicine Hannover, Hanover, Germany

**Keywords:** Bartonella henselae, Bartonella grahamii, Ixodes ricinus, tickborne diseases, vector-borne infections, bacteria, Germany, zoonoses

**To the Editor:** Bacteria of the genus *Bartonella* are transmitted by arthropods and are often implicated in human disease. Even though ticks are known to transmit a variety of pathogens, vector competences for transmission of *Bartonella* spp. by ticks were speculative (*1*) until recently, when in vivo transmission of *B. birtlesii* by *Ixodes ricinus* ticks was demonstrated in mice (*2*). This finding suggests that this tick species, which is common in Europe, may also transmit zoonotic *Bartonella* spp. Evidence of possible tick transmission of bartonellae to humans under natural conditions was provided by Eskow et al. (*3*) and Angelakis et al. (*4*), who identified *Bartonella* spp. in tissue samples of patients who were recently bitten by ticks. We determined the prevalence of *Bartonella* spp. in questing *I. ricinus* ticks in the city of Hanover, Germany, which is nicknamed The Green Metropolis and was selected the German Capital of Biodiversity in 2011.

During April–October 2010, we collected 2,100 questing ticks, consisting of 372 adults (177 female and 195 male), 1,698 nymphs, and 30 larvae, from 10 recreation areas in Hanover. Tick DNA was extracted by using the NucleoSpin 8 Blood kit (Macherey-Nagel, Düren, Germany). Plasmid DNA constructed from *B. henselae* reference strain ATCC49793 containing the 249-bp target sequence of the *gltA* gene was used as positive control. *Bartonella* spp. in ticks was detected by quantitative PCR (qPCR) by using the Mx3005 Multiplex Quantitative PCR System (Stratagene, Heidelberg, Germany) according to the protocol described by Mietze et al. (*5*), with minor modifications. Samples positive by qPCR were verified by gel electrophoresis. *Bartonella* species were differentiated by sequencing (Eurofins MWG Operon, Ebersberg, Germany), and obtained sequences underwent BLAST (http://blast.ncbi.nlm.nih.gov/Blast.cgi) comparison to published sequences.

On the basis of the amplicon-specific melting temperature and DNA bands representing the specific size of 249-bp after gel electrophoresis, results of qPCR showed 100 (4.76%) infected *I. ricinus* ticks (Table). Positive results did not vary by developmental tick stages; 4.84% (18/372) adult ticks (5.08% [9/177] female and 4.62% [9/195] male), 4.71% (80/1,698) nymphs, and 6.67% (2/30) larvae were infected (Table). Because *Bartonella* spp. do not seem to be transmitted transovarially (*6*), it is likely that larvae had interrupted blood meals and thus did not take up enough blood to develop into the nymphal stage.

**Table:** Seasonal distribution of *Ixodes ricinus* ticks infected with *Bartonella* spp., Hanover, Germany, 2010*

Ticks	April	May	June	July	August	September	October	Total
No. infected ticks/no. tested (%)	5/300 (1.67)	38/300 (12.67)	7/300 (2.33)	10/300 (3.33)	5/300 (1.67)	17/300 (5.67)	18/300 (6.00)	100/2,100 (4.76)
No. (%) adults positive	1/88 (1.14)	8/48 (16.67)	0/39	0/41	2/56 (3.57)	3/53 (5.66)	4/47 (8.51)	18/372 (4.84)
No. (%) females	1/32 (3.13)	3/19 (15.79)	0/20	0/17	1/32 (3.13)	2/28 (7.14)	2/29 (6.90)	9/177 (5.08)
No. (%) males	0/56	5/29 (17.24)	0/19	0/24	1/24 (4.17)	1/25 (4.00)	2/18 (11.11)	9/195 (4.62)
No. (%) nymphs	3/203 (1.48)	30/248 (12.10)	7/261 (2.68)	10/259 (3.86)	3/244 (1.23)	14/240 (5.83)	13/243 (5.35)	80/1,698 (4.71)
No. (%) larvae	1/9 (11.11)	0/4	ND	ND	ND	0/7	1/10 (10.00)	2/30 (6.67)

*ND, testing not done.

Seasonal changes in *Bartonella* spp. infection rates resulted in a higher peak in May (38/300 [12.67%]) than in the other months (Table). For sampling locations, infection rates for grassy sampling location 6 (4/210 [1.90%] infected ticks) differed significantly (Bonferroni-Holm adjusted p<0.001; *<0.0011) from that of densely wooded sampling location 9 (22/210 [10.48%] infected ticks).

Sequencing of the *gltA* fragment resulted in *Bartonella* species identification for 56/100 positive samples; 52 of these samples (from 38 nymphs, 13 adults, and 1 larva) were identified as infected with *B. henselae*. In 51 samples (92.86%), maximum identity with the BLAST top hit sequence was 99% because of nucleotide substitutions in position 198 (T→C) and in position 136 (A→G) of the 249-bp fragment. The remaining sample showed 100% identity with *B. henselae* strains Brazil-1 and 45-00249 (GenBank accession nos. HQ012580 and GQ225709).

Four of the 56 successfully sequenced samples (7.14%; all samples from nymphs) showed the sequence pattern of *B. grahamii*. One sample revealed 100% identity with *B. grahamii* (GenBank accession no. EU014266); the remaining 3 samples showed an identity of 98% with the *B. grahamii* strain Hokkaido-1 (GenBank accession no. AB426652) and 99% (T→C in position 93) with a sequence described as *B. grahamii*–like (GenBank accession no. AY435122). Sequences obtained in this study (deposited in GenBank under accession nos. JQ770304 and JK758018) support the genetic variability of *Bartonella* spp., as demonstrated by others (*5*,*7*,*8*).

It remains unclear whether ticks are involved in transmission of pathogenic *Bartonella* spp. to humans under natural conditions. However, the total prevalence rate of 4.76% (100/2,100) questing *I. ricinus* ticks infected with *B. henselae* and *B. grahamii* highlights the need for public awareness and draws attention to the possibility of an infection with zoonotic *Bartonella* spp. after a tick bite (*3*,*4*). *B. henselae*, the predominantly identified species, has been associated with cat scratch disease, peliosis hepatis, and bacillary angiomatosis in humans. Eskow et al. (*3*) also connected chronic symptoms of Lyme disease to co-infections with *Borrelia burgdorferi* and *B. henselae*. *B. grahamii* has been associated with neuroretinitis and ocular artery thrombosis in humans (*9*,*10*). The potential risk for zoonotic *Bartonella* spp. infection in urban recreation areas should not be underestimated.

## References

[1] Billeter SA, Levy MG, Chomel BB, Breitschwerdt EB. Vector transmission of *Bartonella* species with emphasis on the potential for tick transmission. Med Vet Entomol. 2008;22:1–15. 10.1111/j.1365-2915.2008.00713.x 18380649

[2] Reis C, Cote M, Le Rhun D, Lecuelle B, Levin ML, Vayssier-Taussat M, Vector competence of the tick *Ixodes ricinus* for transmission of *Bartonella birtlesii.* PLoS Negl Trop Dis. 2011;5:e1186. 10.1371/journal.pntd.0001186 21655306 PMC3104967

[3] Eskow E, Rao RV, Mordechai E. Concurrent infection of the central nervous system by *Borrelia burgdorferi* and *Bartonella henselae*: evidence for a novel tick-borne disease complex. Arch Neurol. 2001;58:1357–63. 10.1001/archneur.58.9.1357 11559306

[4] Angelakis E, Billeter SA, Breitschwerdt EB, Chomel BB, Raoult D. Potential for tick-borne bartonelloses. Emerg Infect Dis. 2010;16:385–91. 10.3201/eid1603.091685 20202411 PMC3322042

[5] Mietze A, Morick D, Kohler H, Harrus S, Dehio C, Nolte I, Combined MLST and AFLP typing of *Bartonella henselae* isolated from cats reveals new sequence types and suggests clonal evolution. Vet Microbiol. 2011;148:238–45. 10.1016/j.vetmic.2010.08.012 20863631

[6] Cotté V, Bonnet S, Le Rhun D, Le Naour E, Chauvin A, Boulouis HJ, Transmission of *Bartonella henselae* by *Ixodes ricinus.* Emerg Infect Dis. 2008;14:1074–80. 10.3201/eid1407.071110 18598628 PMC2600320

[7] Arvand M, Schubert H, Viezens J. Emergence of distinct genetic variants in the population of primary *Bartonella henselae* isolates. Microbes Infect. 2006;8:1315–20. 10.1016/j.micinf.2005.12.015 16697237

[8] Ehrenborg C, Handley S, Ellis B, Mills J, Holmberg M. *Bartonella grahamii* infecting rodents display high genetic diversity over short geographic distances. Ann N Y Acad Sci. 2003;990:233–5. 10.1111/j.1749-6632.2003.tb07369.x 12860632

[9] Serratrice J, Rolain JM, Granel B, Ene N, Conrath J, Avierinos JF, Bilateral retinal artery branch occlusions revealing *Bartonella grahamii* infection. Rev Med Interne. 2003;24:629–30. 10.1016/S0248-8663(03)00224-8 12951187

[10] Kerkhoff FT, Bergmans AM, van Der Zee A, Rothova A. Demonstration of *Bartonella grahamii* DNA in ocular fluids of a patient with neuroretinitis. J Clin Microbiol. 1999;37:4034–8. 10565926 10.1128/jcm.37.12.4034-4038.1999 PMC85873

